# Revisiting I-BAR Proteins at Central Synapses

**DOI:** 10.3389/fncir.2021.787436

**Published:** 2021-12-16

**Authors:** Christina Chatzi, Gary L. Westbrook

**Affiliations:** Vollum Institute, Oregon Health and Science University, Portland, OR, United States

**Keywords:** BAR domain protein, post-synaptic, synaptic plasticity, dendritic spines, membrane curvature, filopodia

## Abstract

Dendritic spines, the distinctive postsynaptic feature of central nervous system (CNS) excitatory synapses, have been studied extensively as electrical and chemical compartments, as well as scaffolds for receptor cycling and positioning of signaling molecules. The dynamics of the shape, number, and molecular composition of spines, and how they are regulated by neural activity, are critically important in synaptic efficacy, synaptic plasticity, and ultimately learning and memory. Dendritic spines originate as outward protrusions of the cell membrane, but this aspect of spine formation and stabilization has not been a major focus of investigation compared to studies of membrane protrusions in non-neuronal cells. We review here one family of proteins involved in membrane curvature at synapses, the BAR (Bin-Amphiphysin-Rvs) domain proteins. The subfamily of inverse BAR (I-BAR) proteins sense and introduce outward membrane curvature, and serve as bridges between the cell membrane and the cytoskeleton. We focus on three I-BAR domain proteins that are expressed in the central nervous system: Mtss2, MIM, and IRSp53 that promote negative, concave curvature based on their ability to self-associate. Recent studies suggest that each has distinct functions in synapse formation and synaptic plasticity. The action of I-BARs is also shaped by crosstalk with other signaling components, forming signaling platforms that can function in a circuit-dependent manner. We discuss another potentially important feature—the ability of some BAR domain proteins to impact the function of other family members by heterooligomerization. Understanding the spatiotemporal resolution of synaptic I-BAR protein expression and their interactions should provide insights into the interplay between activity-dependent neural plasticity and network rewiring in the CNS.

## Introduction

Bin-Amphiphysin-Rvs (BAR) domain proteins have recently taken center stage as specialized effector proteins coordinating actin cytoskeleton and membrane remodeling and facilitating cell-signaling events. Membrane remodeling and cell shape reorganization in response to signals from neighboring cells or the environment are essential functions of all cells including neurons. In neurons, such modulation of membrane topology is necessary for axonal and dendritic morphogenesis, generation of presynaptic sites of neurotransmitter release, and the initiation and maintenance of the protrusions known as dendritic spines at central excitatory synapses. BAR domains form anti-parallel dimers, each consisting of three bent anti-parallel alpha helices as well as two protruding arms (Peter et al., 2004). BAR-domain-containing proteins can be divided into three categories based on their differential membrane curvature: BAR/N-BAR, F-BAR, and I-BAR. BAR/N-BAR and F-BAR proteins have been well described in synaptic vesicle endocytosis, recycling, and structural organization of synapses (Simunovic et al., 2019) The CNS role of the third category, the I-BAR protein subfamily, the focus of this review, has been much less explored.

BAR/N-BAR domain dimers have a crescent shape and promote inward convex membrane curvatures. The prototype of this category is amphiphysin, which is highly expressed in mammalian neurons and critical to endocytosis of synaptic vesicles at presynaptic nerve terminals (Qualmann et al., 2011; Kessels and Qualmann, 2015). A second large category contains an F-BAR (Fes/CIP4 homology BAR) domain. The atomic structure of F-BAR domains show a less curved, more elongated shape compared to classical BAR domains, with five alpha helices followed by a short sequence for homodimerization (Henne et al., 2007; Shimada et al., 2007). Most F-BAR proteins are involved in endocytic and vesicle trafficking events in neuronal morphogenesis and migration (Itoh et al., 2005; Dharmalingam et al., 2009; Hartig et al., 2009; Feng et al., 2010). In contrast, I-BAR and inverse F-BAR (iF-BAR) protein subfamilies promote concave curvatures, as found in slit-Robo GTPase-activating proteins (srGAP 1-4), which localize along negatively curved membranes and regulate the development and maturation of excitatory and inhibitory synapses (Saarikangas et al., 2008, 2015; Carlson et al., 2011; Endris et al., 2011; Charrier et al., 2012; Coutinho-Budd et al., 2012; Waltereit et al., 2012; Bacon et al., 2013; Fossati et al., 2016; Chatzi et al., 2019).

We discuss here recent findings regarding the role of I-BAR proteins in synaptic formation and in structural and functional plasticity. Accumulating evidence indicates that I-BAR proteins are involved in excitatory synapse formation and post-synaptic plasticity including the induction, shaping, and morphological remodeling of dendritic spines and anchoring and trafficking of post-synaptic receptors (Hori et al., 2005; Kim et al., 2009; Sawallisch et al., 2009; Burette et al., 2014; Saarikangas et al., 2015; Dosemeci et al., 2017; Sistig et al., 2017; Kawabata Galbraith et al., 2018; Chatzi et al., 2019; Minkeviciene et al., 2019). I-BAR-containing proteins have actin-binding domains that synergize with actin to form filopodia and dendritic spines (Hori et al., 2005; Kim et al., 2009; Sawallisch et al., 2009; Burette et al., 2014; Saarikangas et al., 2015; Dosemeci et al., 2017; Sistig et al., 2017; Kawabata Galbraith et al., 2018; Chatzi et al., 2019; Minkeviciene et al., 2019). The morphological changes associated with the organization and dynamics of the synaptic actin cytoskeleton regulate activity-dependent synaptic plasticity including the formation of new synapses as well as remodeling of existing connections. This plasticity represents a key mechanism for rewiring neural circuits during development, learning and memory, and in brain disorders (Stuchlik, 2014; Khanal and Hotulainen, 2021).

### The Generation of Membrane Protrusions by I-BAR Domains

The I-BAR domain was initially identified as a homologous domain in the N-terminus of two mammalian proteins, Insulin Receptor Substrate of 53 kDa (IRSp53) and Missing in Metastasis (MIM), and thus labeled as an IM domain (IRSp53/MIM; Zhao et al., 2011). Because of structural similarity to classical BAR domains, the IM domain was subsequently renamed as the I-BAR (Inverse-BAR) domain (Scita et al., 2008). Interestingly, three of the five mammalian I-BAR proteins are expressed in the CNS: IRSp53, MIM (or Mtss1, Metastasis suppressor 1), and ABBA/Mtss2 (actin-bundling protein with BAIAP2 homology, previously Mtss1L). We will refer to these three proteins as IRSp53, MIM, and Mtss2, respectively. As for the other two mammalian I-BARs, brain expression of IRTKS’ (insulin receptor tyrosine kinase substrate; also known as BAIAP2L1) is low whereas little is known about BAIAP2L2 (FLJ22582; Millard et al., 2007).

**Figure 1:**
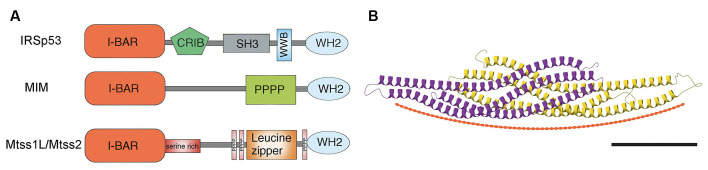
**(A)** Schematic of domain structures of mammalian I-BAR domain proteins. I-BAR: I-BAR domain, WH2: WH2 domain, SH3: SH3 domain, PPPP: proline-rich region, Serine rich: serine-rich region, CRIB: Cdc42- and Rac-interactive binding domain, and WWB: WW binding domain. Modified from Sistig et al. (2017). **(B)** Structural and curvature-inducing activity of the I-BAR domain. Shown is an X-ray crystal structure of Mtss2 with each monomer color-coded. Scale bar: 5 nm. Modified from Abou-Kheir et al. (2008).

Unlike the substantial structural variability among members of the BAR/N-BAR and F-BAR subfamilies, I-BAR subfamily members are closely related. They share the N-terminal I-BAR domain, which binds to membranes and promotes self-association resulting in head-to-head dimers with their C-terminal protruding at each end. The I-BARs contain other protein-protein interaction domains that can contribute to specific signaling complexes in different cellular environments (Figure 1A). For example, IRSp53 has an SH3 domain, which binds to proline-rich sequences in regulators of actin dynamics, such as WAVE-2, Mena, Eps8, PSD-95, and Shank; a CRIB (Cdc42- and Rac-interactive binding) domain in its C-terminal interacts with the small GTPase Cdc42; as well as a PDZ binding domain (Miki et al., 2000; Krugmann et al., 2001; Funato et al., 2004; Soltau et al., 2004; Abou-Kheir et al., 2008). MIM and Mtss2 can associate with each other, incorporating an actin monomer-binding WH2 (WASP-homology 2) domain at the C-terminus, which suggests they function at the interface between the plasma membrane and the actin cytoskeleton (Zhao et al., 2011). Despite their closely related I-BAR domain, MIM and Mtss2 differ in several other domains (Figure 1A). For example, Mtss2 has a serine-rich domain that suggests a role of phosphorylation and a leucine zipper domain that could affect dimerization (Zhao et al., 2011). These sorts of regulatory effects and interactions between family members have yet to be fully explored.

Structurally, the I-BAR domains assume a zeppelin-like structure with two alpha helical anti-parallel dimers (Saarikangas et al., 2009; Zhao et al., 2011; Bassereau et al., 2018; Figure 1B). The I-BAR domain induces plasma membrane protrusions by binding the inner leaflet of phosphoinositide-rich membranes through a lipid-binding surface, resulting in tubular structures with convex geometry and thus inducing negative membrane curvature (Saarikangas et al., 2009; Zhao et al., 2011). MIM and Mtss2 have N-terminal amphipathic helices, which result in tubules with diameters (~70 nm), which are larger than those made by IRSp53 (Saarikangas et al., 2009). Whether this difference is important in dendritic spine size is not clear. I-BAR domains induce strong PI(4,5)P2 clustering upon membrane binding, modulating their lipid organization (Saarikangas et al., 2009; Zhao et al., 2011). MIM initiates the formation of spine proto-protrusions in a PI(4,5)P2-dependent manner *in vitro*. PIPs serve as good candidates to recruit spine-initiating factors to nascent synapses, however, the mechanism by which they are involved in spine initiation remains unclear. *In vitro* MIM-GFP localizes to dendrites with higher expression in spine heads and filopodia (Saarikangas et al., 2015). In general, I-BAR oligomerization increases membrane binding, which might correspond with lipid microdomains. Such an organization may act as structural platforms for the recruitment of other proteins and the formation of local signaling centers. Whether this is cause or result is not yet known.

### Expression Patterns and Localization of I-BAR Proteins in the Postnatal Brain

Given the tubulating activity of I-BAR domains and their helical assembly, the expression patterns of each I-BAR family member in the brain and their subcellular localization is critical to understanding their function in dendritic spines and synaptic function. Of the three I-BAR proteins expressed in the CNS, IRSp53, the founding member of the subfamily, has been the most extensively studied. IRSp53 mRNA is abundantly expressed in brain regions rich in spiny neurons including the hippocampus, cerebellum, striatum, and cortex (Abbott et al., 1999; Bockmann et al., 2002; Burette et al., 2014). The protein distribution of IRSp53, assessed by X-gal staining in brain slices from *IRSp53*^+/−^ reporter mouse brains and by immunohistochemistry of endogenous IRSp53 in rat brains, follows a similar pattern (Bockmann et al., 2002; Kim et al., 2009; Sawallisch et al., 2009; Burette et al., 2014). Expression reaches a maximum during the first 3 weeks postnatal, a period of active synaptogenesis (Bockmann et al., 2002; Choi et al., 2005). In the adult mouse hippocampus, IRSp53 immunoreactivity is present in the dentate gyrus and CA1 as well as in CA2 and CA3 (Burette et al., 2014). Immunolabeling is present in the soma and apical dendrites of dentate granule cells and in pyramidal neurons (Burette et al., 2014). The punctate immunolabeling throughout the neuropil in the molecular layer of the dentate gyrus, and the stratum radiatum and stratum oriens in CA1 are consistent with synaptic localization on dendritic-spine bearing neurons (Burette et al., 2014). Somata and proximal dendrites of Purkinje cells in the cerebellum, one of the most “spiny” neurons in the brain, also exhibit immunopositive puncta in the molecular and granule cell layers (Burette et al., 2014). IRSp53 staining is prominent in GABAergic medium spiny neurons in the striatum whereas it is lacking in aspiny GABAergic interneurons in the neocortex and hippocampus (Burette et al., 2014). Although IRSp53 expression seems to be limited to spiny neurons regardless of their neurotransmitter phenotype; more studies are necessary to verify if that is the case for other members of the I-BAR family.

IRSp53 was the first I-BAR protein to be identified in the postsynaptic density (PSD), the protein complex lining the postsynaptic membrane. The PSD comprises a 30–40 nm thick electron-dense core layer and a deeper, contiguous pallium layer at excitatory synapses (Abbott et al., 1999; Dosemeci et al., 2016). Biochemical fractionation and Western blotting also support the postsynaptic localization of IRSp53. Ultrastructural studies have confirmed the localization of IRSp53 at the PSD as well as in other dendritic spine sub-compartments away from the PSD including tangential walls and the cytosol (Choi et al., 2005). In cultured hippocampal cells, depolarization (by NMDA receptor stimulation or increased extracellular potassium) promotes synaptic translocation of IRSp53, which is dependent on PKC-dependent phosphorylation of the I-BAR domain and the PDZ-binding domain (Hori et al., 2005). This observation suggests that the localization of IRSp53 can be regulated by neuronal activity (Hori et al., 2005). Interestingly, IRSp53 within the PSD differs between brain regions. For example, in excitatory neurons in the neocortex and hippocampus, IRSp53 is concentrated at the center of the PSD, whereas in spiny inhibitory neurons of the neostriatum and cerebellar cortex it is distributed more uniformly (Burette et al., 2014). As dendritic spines can differ in ultrastructure, protein composition, and signaling pathways (Harris and Weinberg, 2012; O’Rourke et al., 2012), the specific localization of I-BAR proteins within the dendritic spine may provide clues to molecular organization and function.

For MIM, *in situ* hybridization, western blotting, and immunohistochemical analyses show neuronal-specific expression in the adult hippocampus and the cerebellum (Saarikangas et al., 2015; Sistig et al., 2017). In the hippocampus, MIM immunoreactivity is localized in soma and dendrites of pyramidal cells in CA1 and CA3 and dentate granule cells. MIM expression is highest in Purkinje cells where it reaches a maximum at P8 (Holst et al., 2008; Sistig et al., 2017). At this early postnatal stage, its expression is restricted to the soma whereas by adulthood it is expressed throughout the dendrites (Kawabata Galbraith et al., 2018; Minkeviciene et al., 2019). Cerebellar granule cells also express MIM early in development, but expression decreases after P15 (Holst et al., 2008). MIM is absent in axons of hippocampal and cerebellar neurons supporting a largely postsynaptic location in dendrites (Saarikangas et al., 2015). MIM expression has been reported across different ages in the mouse cortex, hippocampus, and the cerebellum with the highest expression in Purkinje cells across the lifespan, but in some reports expression in cortex and hippocampus was only detected in early development (P7; Minkeviciene et al., 2019).

Mtss2 (formerly known as Mtss1L or ABBA) has been the least studied I-BAR protein in the CNS. Initially, Mtss2 was detected in radial glial cells during development and implicated in glial membrane protrusions and end-feet (Saarikangas et al., 2008). However, recent evidence indicates neuronal expression of Mtss2 that is activity-dependent in mature granule cells of the hippocampal dentate gyrus in adult mice (Chatzi et al., 2019). Thus, neuronal expression of Mtss2 may have been underappreciated because of its unrecognized activity-dependent expression. For example, in mouse adult dentate granule cells, Mtss2 mRNA is upregulated in granule cells activated by a single bout of voluntary exercise. In Mtss2 KOMP reporter mice (Chatzi et al., 2019) in which the endogenous *Mtss2* promoter drives bacterial beta-galactosidase (*lacZ*) LacZ expression was undetectable at baseline, then peaked at 3 days post-exercise parallel with mRNA expression (Saarikangas et al., 2008). In the cerebellum, Purkinje cells with their high basal firing rates are the only neurons that express Mtss2 at baseline in adult mouse brain (Chatzi, unpublished data). Endogenous Mtss2 immunoreactivity in cultured hippocampal neurons could be detected only after exposure to brain-derived neurotrophic factor (BDNF), which is upregulated by exercise and involved in activity-dependent synaptic plasticity (Chatzi et al., 2019). *In vitro* Mtss2 also colocalizes with the somatodendritic marker MAP2 along dendrites and in dendritic spines. As Mtss2 had not been previously recognized as activity-dependent, it will be important to investigate its expression at different ages and in other cell types such as radial glial and interneurons. Examining how activity-dependent induction of I-BAR signaling cascades in different cells types impact synaptic and circuit function will be important for understanding plasticity and remodeling of synapses.

The expression patterns of IRSp53, MIM, and Mtss2 overlap in the somatodendritic compartment of neurons in the cerebellum and the hippocampus, but how that relates to synaptic function is not yet clear. A remaining challenge to understanding their functional significance will be to distinguish their cytosolic pools, which might represent protein in transit (unbound vs. interacting with other cytoskeletal elements) from the membrane-associated pool in and around dendritic spines. It is possible that the cytosolic pool in the dendritic shaft represents a reserve pool that can translocate to spines and initiate spine formation by binding to membranes, thus acting as an early effector of synapse formation. I-BAR signaling cascades elicited by synaptic stimulation could be synapse-specific, thus representing biochemical signaling compartments that confine structural plasticity to individual spines and circuits. Compartmentalization of I-BAR proteins could facilitate synapse-specific plasticity and thereby regulate the strength of synaptic connections. To date, it has not been possible to directly assess these issues because the limited resolution of live-cell light microscopy has not allowed a direct correlation of subcellular localization and dynamics of I-BAR proteins to be established. Emerging technologies in fluorescence microscopy that enable imaging at resolutions below the diffraction limit in combination with tagging of I-BAR proteins *in vitro* and *in vivo* may now make it possible to determine how spine initiation and plasticity affects localization and diffusion of cytoplasmic or membrane-associated I-BAR proteins.

### I-BAR Proteins in Dendritic Spine Development and Synaptic Plasticity

Dendritic spines serve as the hubs for the organization of excitatory synapses. These membrane protrusions coordinate dynamic interactions between membrane shaping proteins, scaffolding molecules, receptors, signaling molecules, and, importantly, the actin cytoskeleton (Tonnesen and Nagerl, 2016). Actin remodeling is an essential component of spine development and plasticity (Hotulainen and Hoogenraad, 2010). Both MIM and Mtss2 contain the actin monomer binding WH2-domain that could facilitate actin polymerization (Zhao et al., 2011). However, the WH2 domain of MIM was not needed for filopodia formation or the increase in spine density with MIM overexpression (Saarikangas et al., 2015). Initiation of dendritic spines by direct membrane bending by the MIM I-BAR domain *in vitro* critically required actin filament assembly by the Arp2/3 complex (Saarikangas et al., 2015). The Arp2/3 complex is activated by *nucleation-promoting factors* WASP and WAVE that are in turn activated by phosphoinositides, Rho GTPases, and BAR-domain proteins (Soderling, 2009). Thus I-BAR-PI(4,5)P2 clustering could recruit Arp2/3 complex activators WASP and WAVE to spine initiation sites. Interestingly the nucleation-promoting factor WASP co-localized with MIM at spine initiation sites to form filopodia *in vitro*. Recent results suggest that WAVE1 induces sheet-type actin polymerization, whereas N-WASP creates more focal point polymerization (Pipathsouk et al., 2021). Differential interactions of I-BAR domains with WASP may facilitate actin polymerization in dendritic filopodia whereas interactions with WASP might be needed for more organized actin structures such as spine heads. Interestingly in cerebellar Purkinje cells, MIM can also bind to the formin DAAM1 and inhibit the actin polymerization (Kawabata Galbraith et al., 2018). Formin family proteins form straight actin filaments instead of branched actin filament networks (Firat-Karalar and Welch, 2011), both of which can exist in synaptic structures. Thus I-BAR proteins may have multiple functions in local actin regulation during dendritic spine formation.

**Figure 2:**
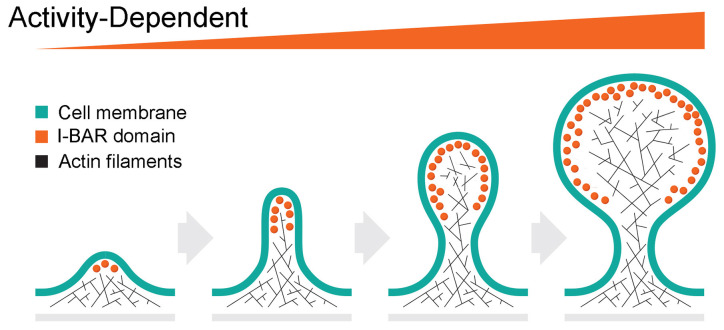
Hypothetical role of I-BAR proteins on activity-dependent spine initiation, elongation and maturation. The formation of the dendritic spine begins with the initiation of filopodia. Activity-dependent recruitment of I-BAR proteins stimulates actin cytoskeleton growth resulting in further elongation of the filopodia. Further increase of I-BAR membrane density induces branching of the actin filaments due to Arp 2/3 complex activity, creates the spine head, and enhances spine plasticity.

The shape of dendritic spines governs, at least in part, their function, ranging from filopodia-like protrusions, some of which are spine precursors, to more mature stubby, thin, or mushroom-shaped structures (Li and Sheng, 2003; Runge et al., 2020). Filopodial facilitate the interaction of presynaptic and postsynaptic membranes during synaptogenesis. Filopodia are dynamic and change shape within a very short time scale (min) (Li and Sheng, 2003; Alimohamadi et al., 2021). Activity-dependent morphological changes also occur in mature spines associated with synaptic plasticity (Li and Sheng, 2003; Alimohamadi et al., 2021). The architecture and activity-dependent plasticity of dendritic spines and their postsynaptic densities (PSDs) enable the tuning of synaptic strength. Given the properties of I-BAR proteins, they are well-positioned to modulate synaptic actin cytoskeleton and membrane substructures during dendritic spine formation and synaptic plasticity (Figure 2).

Consistent with this idea, gain- and loss- of function phenotypic analyses of the IRSp53 and MIM have revealed potential roles in synapse formation (Choi et al., 2005; Kim et al., 2009; Sawallisch et al., 2009; Burette et al., 2014; Saarikangas et al., 2015; Sistig et al., 2017; Chatzi et al., 2019; Minkeviciene et al., 2019). IRSp53 expression can affect the number as well as the functional properties of excitatory synapses (Kang et al., 2016). For example, downregulation of IRSp53 in cultured hippocampal neurons decreased the density, but not size, of dendritic spines whereas overexpression increased spine density and size (Choi et al., 2005). Similarly, excitatory synapse formation is delayed in IRSp53^−/−^ hippocampal neurons *in vitro* (Kim et al., 2009; Sawallisch et al., 2009). Interestingly, these knockout mice did not show alterations in excitatory synapse formation in the hippocampus *in vivo* (Kim et al., 2009; Sawallisch et al., 2009), but were reported to increase NMDA receptor-mediated synaptic responses and enhance long-term potentiation as well as hippocampal-dependent learning deficits. These authors hypothesized that IRSp53 deletion leads to an abnormal stabilization of synaptic actin filaments, which may promote synaptic localization of NMDARs under basal conditions and suppress their activity-dependent relocation (Chung et al., 2015). In contrast, in the medial prefrontal cortex (mPFC), IRSp53^−/−^ mice show a decrease in frequency and amplitude of mEPSCs and synapse numbers by EM without affecting NMDA receptor function (Chung et al., 2015). Deletion of IRSp53 in glutamatergic neurons and GABAergic mPFC neurons lead to distinct behavioral deficits and synaptic changes between male and female mice (Kim et al., 2020). Whether these differences reflect alterations at the synaptic or circuit level or reflect compensation by other I-BAR proteins is not yet clear. However, it seems plausible that I-BAR proteins may act not only as initiators of membrane protrusions but also as adaptors in signaling complexes.

Synaptic phenotypes are also apparent in MIM knockout mice (MIM KO). Cerebellar Purkinje cells show the most striking phenotype with a reduced number of spines and abnormal dendritic morphology (Saarikangas et al., 2015; Sistig et al., 2017). These abnormalities correlate with deficits in motor coordination and altered electrophysiological properties in Purkinje cells. Interestingly, in the hippocampus the total spine density in CA1 pyramidal neurons of adult MIM KO mice was unaffected, however, there was a slight reduction in thin and stubby spines (Minkeviciene et al., 2019). Despite the modest changes in spine density, MIM KO mice exhibit hippocampal-dependent spatial learning defects and decreased anxiety-like behavior in the water maze and open field tests similar to IRSp53 KO mice. It is possible that compensation by IRSp53 in the absence of MIM is sufficient for synapse formation in the hippocampus, but proper brain function nevertheless requires the integrative presence of both proteins in the post-synaptic compartment.

Similar to MIM, PI(4,5)P2-dependent membrane localization is necessary for iF-BAR protein SrGAP3 to induce filopodia-like protrusions *in vitro* (Carlson et al., 2011). Whereas both SrGAP3 and MIM proteins support the initiation of thin spines, the former also inhibits the transition of thin spines to mushroom spines (Carlson et al., 2011; Saarikangas et al., 2015). Similarly, the iF-BAR domain of srGAP2 promotes filopodial formation in cortical neurons (Coutinho-Budd et al., 2012). Global knockout of SrGAP3 decreases the number of dendritic filopodia during early mouse development (Carlson et al., 2011), whereas loss-of-function of srGAP2 results in increased spine density with delayed spine maturation (Charrier et al., 2012).

The role of Mtss2 in spine density and synaptic function has been largely unexplored. Our recent work identified activity-dependent expression of Mtss2 as a novel effector of synaptic rearrangement. Overexpression of Mtss2 in hippocampal neurons increased dendritic spine density *in vitro* and *in vivo*, consistent with a role of its I-BAR domain in membrane curvature and dendritic spine formation (Chatzi et al., 2019). A single episode of voluntary exercise increased neural activity in a subset of dentate granule cells. The activated cells showed a laminar-specific increase in excitatory EPSCs and dendritic spines in the outer molecular layer that receives input from the lateral entorhinal cortex, whereas inputs on the same dendrites from the medial entorhinal cortex were unaffected. shRNA-mediated Mtss2 knockdown *in vivo* prevented these changes (Chatzi et al., 2019), suggesting postsynaptic targeting of Mtss2 mRNA or protein to activated synapses. These changes peaked at 3 days post-stimulus, a time course consistent with activity-dependent rearrangement of the synaptic circuit. Given the presence of other I-BARs at these synapses, it is possible that there is a coordinated action of multiple I-BAR proteins for activity-dependent plasticity of dendritic spines. The spatial and temporal profile of these proteins in synaptic plasticity maintenance and brain function is intriguing, given the potential interactions of the I-BAR subfamily by heteroligomerization. For example, overexpression *in vitro* recruits MIM to the dendritic membrane in a PIP_2_-dependent manner, resulting in its oligomerization and the induction of dendritic filopodia, often precursors of dendritic spines (Saarikangas et al., 2015).

### I-BAR Heteroligomerization and Inhibition

Given the presence of three I-BAR proteins at synapses, it is intriguing to consider whether BAR-BAR heteroligomerization has a role in dendritic spine formation and synaptic plasticity. BAR domain proteins usually associate with a superfamily member with a similar domain organization. However, it is unclear whether this association occurs through heterodimerization or side-by-side interaction of homodimers. For example, heterodimeric BAR domain complexes of the islet cell autoantigen of 69 kDa (ICA69) with PICK1 regulate AMPA receptor trafficking and prevent the formation of PICK1 homomeric complexes (Cao et al., 2007). Oligomers between similar BAR domain proteins can also modulate their function (Charrier et al., 2012). The iF-BAR domain of srGAP2 (srGAP2C) occurs naturally in humans and inhibits SRGAP2 by dimerization (Charrier et al., 2012). Thus, expression of SRGAP2C results in phenotypes similar to SRGAP2 deficiency, including neoteny during spine maturation and increased density of longer spines (Charrier et al., 2012). Such interactions have not yet been reported for I-BAR subfamily members, but their combinatorial association could considerably expand their role in synapse formation and postsynaptic plasticity.

## Summary

Although the role of BAR proteins in membrane curvature is well known in non-neuronal cells, emerging evidence suggests that the I-BAR subfamily expressed in the central nervous system have an important role in the formation and maintenance of dendritic spines at excitatory synapses. The activity-dependence of Mtss2 expression also raises the possibility that the I-BAR subfamily is directly involved in structural and functional plasticity that underlies both developmental events as well as activity-dependent reorganization of synaptic networks. The overlapping expressions of the three I-BAR proteins in the hippocampus and other neuronal circuits begs the question of what mechanisms regulate their precise recruitment to the right synapses and at the right time. One possibility is that homodimerization and heterodimerization between Mtss2 and MIM/IRSp53 could act as activity-dependent on-off switches respectively.

Critical to assessing these possibilities are experiments to address input-specific plasticity—synaptic tagging for differential subcellular localization of I-BARs. New tools have to be developed to bridge the gap between molecular, biochemical, and structural observations *in vitro* and *in vivo*. For example, recent advances in cell biology with controlled expression levels of protein tags (CRISPR/Cas9) together with super resolution microscopy and 3D imaging should allow quantification and subcellular localization in the future of the densities of I-BAR proteins revealing their role in synaptic function.

## Author Contributions

CC and GW contributed equally to the main conceptual ideas and writing of the manuscript. All authors contributed to the article and approved the submitted version.

## Conflict of Interest

The authors declare that the research was conducted in the absence of any commercial or financial relationships that could be construed as a potential conflict of interest.

## Publisher’s Note

All claims expressed in this article are solely those of the authors and do not necessarily represent those of their affiliated organizations, or those of the publisher, the editors and the reviewers. Any product that may be evaluated in this article, or claim that may be made by its manufacturer, is not guaranteed or endorsed by the publisher.
